# Getting Inside Closed‐Loop Referrals: Exploring the Patient Experience of Finding and Connecting to Social Care With a Community Resource Referral System Using a Community‐Based Participatory Approach

**DOI:** 10.1111/1475-6773.14451

**Published:** 2025-02-16

**Authors:** Anthony W. Olson, Nik Allen, Ardem Elmayan, Kim Green, Melissa L. Harry, Jenny Kempfert, Emily Kuenstler, Heath Maki, Sarah Nelson, Mary Rapps, Rose St. John, Salaam Witherspoon, Shawandala Brown, Ashlie Castaldo, Melissa Grimes, Treasure Jenkins, Kennedy Mosher

**Affiliations:** ^1^ Essentia Institute of Rural Health Duluth Minnesota USA; ^2^ University of Minnesota, College of Pharmacy Duluth Minnesota USA; ^3^ Lincoln Park Children & Families Collaborative Duluth Minnesota USA; ^4^ Arc Northland Duluth Minnesota USA; ^5^ Generations Health Care Initiatives Duluth Minnesota USA; ^6^ Community Action Duluth Duluth Minnesota USA

**Keywords:** health equity, patient assessment/satisfaction, qualitative research, referrals and referral networks, rural health, social determinants of health

## Abstract

**Objective:**

To explore the experiences of patients who found and/or connected to social care via a community resource referral system named “Resourceful” (linked with FindHelp.org) linked to a health system's electronic health record.

**Study Setting and Design:**

The mixed‐methods study was co‐designed and conducted using community‐based participatory processes by a team of researchers at a multi‐state health system (Minnesota, North Dakota, Wisconsin) and community members with lived experience addressing unmet social needs. Study participants were individuals referred to, connected to, or delivered social care through Resourceful in the health system's service area. Quantitative surveys were emailed to 780 patients and 38 healthcare workers (HCWs) tied to closed‐loop referrals between 8/2022 and 2/2023. Qualitative interview invites were emailed to 19 patient survey respondents wanting to interview, and the five HCWs and 12 community‐based organizations (CBOs) involved in their social care experience. Descriptive statistics analyzed sociodemographic and patient experience variables developed via the participatory process. We coded qualitative data using thematic analysis, extracting thematic factors informing survey responses.

**Data Sources and Analytic Sample:**

We collected primary data from online surveys and semi‐structured phone interviews.

**Principal Findings:**

Usable survey responses from 62 patients and 14 HCWs were analyzed. Most respondents agreed on some level that their experience using Resourceful strengthened patient trust (patients:66%, HCWs:86%), improved communication about what patients need to be healthy (patients:61%, HCWs:57%), provided “helpful help” reducing social care barriers (patients:56%, HCWs:93%), and enhanced person‐centeredness (patients:60%, HCWs:79%). Qualitative analysis yielded six thematic factors corresponding to 23 actionable takeaways potentially important for using CRRS to improve the seeker experience of social care: resource/service environment; platform access/usability/utilization; helper integration/coordination/continuity; helpful help; reliable sources/partnerships; responsive relationships.

**Conclusions:**

Differences in the perceptions of patient experiences involving resourceful were observed between patients and HCWs/CBOs. Thematic factors clarified these differences and how to improve patient experiences with closed‐loop referrals.

**Summary**

- What is known about the topic
  ○ Integration of electronic health records (EHR) with community resource referral systems (CRRS) is a growing practice among health systems to connect patients with health‐related social needs (HRSNs) to social care.
  ○ EHR‐integrated CRRS show promise for improving social and healthcare service coordination but also introduce risks for distorting meaningful impacts on health equity and unintended harm.
  ○ Community‐based participatory research and evaluation (CBPE) is an approach for co‐designing, conducting, and disseminating studies while building trust and consensus among individuals and organizations partnering to advance health equity.
- What this study adds
  ○ This 30‐month CBPE study found that most patients using the CRRS to find and/or connect to social care reported a positive experience.
  ○ Health care worker (HCW) helpers reported more positive perceptions than seekers on 8 of 11 seeker experience measures co‐developed by researchers and community members with lived experience addressing unmet HRSNs.
  ○ Several literature‐supported and actionable takeaways for improving patient experiences with CRRS, like incorporating seeker experience measures into CRRS closed‐loop referral reports and exploring healthcare/CBO incentive alignments for high‐quality social care.

## 1 Introduction

Social care attempts to address unmet health‐related social needs (HRSN), which are an individual's adverse social conditions like unstable housing, food insecurity, and unreliable transportation that cause and exacerbate poor health [1, 2, 3, 4]. Without assistance, “seekers” (i.e., patients seeking to address their unmet HRSN) [5] may navigate a complicated maze of organizations, information, processes, and administrative burdens to find and get needed help. Seekers, especially those in crisis, are unlikely to have the time, skills, or other resources to navigate the maze alone, and may experience increased burdens and harm when attempting to find and/or connect to help [6, 7, 8]. Similarly, “helpers” (e.g., healthcare workers [HCWs] and individuals affiliated with community‐based organizations [CBOs]who assist seekers find, connect to, or provide social care) [5] face challenges like time, technology, and information barriers when helping seekers [9, 10, 11]. The result is conceptual and technical silos preventing seekers and helpers from successfully coordinating resources and care [12, 13, 14].

Community Resource Referral Systems (CRRS), also known as resource referral networks or technology, are digital platforms for streamlining how seekers find and/or connect to help to address unmet HRSNs [15, 16, 17]. CRRS capabilities vary by vendor with some systems supporting bi‐directional communication and coordination among seekers and helper organizations [16, 18]. Many health systems are integrating their electronic health records (EHR) with CRRS and developing “closed‐loop referral” processes [16, 17, 18, 19].“Closed‐loop referrals” commonly involve clinicians initiating social care referrals for their patients who screen positive for unmet HRSN, and then receive status updates or other clinically relevant information back from the referred to CBOs. For example, a CRRS enables a clinician seeing a patient who has diabetes, food insecurity, and transportation barriers to use their EHR to auto‐generate three different social care referral emails received by CBOs. The first referral leads to the patient working with the referred to CBO to successfully acquire SNAP benefits. The second referral to a food bank results in a missed call by the patient from the referred to CBO. The third referral ends with the patient informing the referred to CBO that they no longer needed transportation vouchers for food and medical care because a neighbor fixed their car. In this example, text or emails would be sent to the clinician, CBOs, and patient with a link to the CRRS webpage containing the outcomes of the referrals: “got help,” “couldn't be contacted,” and “no longer interested in help” [20]. The CRRS may also include additional details about the cause or context surrounding the referral outcome.

CRRS with closed‐loop referral capabilities can provide important information for coordination but may miss critical information about problems the seeker experienced finding or connecting to help. The result can lead to broken trust and harm to communities at higher risk for experiencing unmet HRSNs [21, 22, 23, 24]. For example, the CRRS may be used to report a seeker “got help” from a CBO but misses key details about the seeker experience, like barriers the seeker overcame (e.g., rescheduled appointment from missing documentation), how seekers felt along the way (e.g., lost/unimportant, hopeful/supported), and how beneficial the help was to seekers (e.g., reduced anxiety, saved time/energy). The example represents important gaps in the understanding of what is important to seekers and helpers using CRRS to address unmet HRSNs. Filling these knowledge gaps can inform implementation, evaluation, and quality improvement.

In 2019, a coalition of CBOs, local foundations, and health systems in Duluth, Minnesota, sought to improve health equity in their service areas by collectively adopting a CRRS. Two years later, the coalition launched a free and publicly available web‐based platform built on the Findhelp Social Care Network [25] (previously Aunt Bertha) named “Resourceful” (WeAreResourceful.org). Resourceful is integrated with a local health system's EHR and assists HCW and CBO helpers in initiating and tracking closed‐loop social care referrals for patients [26]. In this study, we aimed to explore the experiences of patients who found and/or connected to social care through the Resourceful CRRS platform. Our objectives were to: (1) identify what experts with lived experience as seekers, HCW helpers, or CBO helpers view as important areas to evaluate the seeker experience; (2) compare seeker and HCW helper perceptions of the seeker experience; and (3) identify factors that seekers, HCW helpers, and CBO helpers viewed as tied to seeker experiences in closed‐loop referrals.

## 2 Methods

This preplanned, experiential phenomenological mixed methods study followed COREQ guidelines and a community‐based participatory research and evaluation approach (i.e., experts with lived experience partner with formally‐trained researchers to co‐design, conduct, and disseminate the study) [27, 28]. This systematic process can facilitate trust‐building and consensus among individuals and organizations partnering to advance health equity [29]. Fawcett and colleagues developed a six‐step community‐based participatory evaluation (CBPE) framework described in Table 1 [27] applied in this study. The framework was used to embed and prioritize the values, ownership, and views of experts with lived experience addressing unmet HRSN alongside those of researchers, health systems, and CBOs.

**TABLE 1:** Community‐based participatory evaluation step descriptions and completion dates [27].

Steps	Description (# of CAT members involved)	Step duration
1. Naming and framing of goals	Identify shared goals for evaluating success (16: AC/AWO/EK/HM/JK/KG/MG/MR/NA/RSJ/SB/SD/SN/SW)	August 2021–December 2021
2. Develop a logic model for success	Backwards plan the information, people, activities, measures, and other needs for the evaluation (15: AWO/EK/HM/JK/KG/KM/MG/MLH/MR/NA/RSJ/SB/SN/SW/TJ)	January 2022–May 2022
3. Focus the evaluation	Identify the specific questions about that will be used to evaluate the success of Resourceful (15: AWO/EK/HM/JK/KG/KM/MG/MLH/MR/NA/RSJ/SB/SN/SW/TJ)	June 2022–November 2022
4. Gather information and evidence	Collect information and evidence to evaluate the success of Resourceful (9: AE/AWO/KG/MLH/MR/NA/RSJ/SN/SW)	December 2022–June 2023
5. Analyze information	Analyze the information and evidence to evaluate the success of Resourceful (5: AE/AWO/KG/SN/SW)	July 2023–October 2023
6. Share and celebrate success	Identify how the findings from Step 5 will be used and shared to improve resourceful	November 2023–January 2024^a^

^a^ The completion date of Step 6 is identified as January 2024, when funding from study sponsors ended, but community advisory team (CAT) members have continued to propose forums to share study findings.

The study was co‐designed and conducted by a community advisory team (CAT) composed of experts with lived experience as seekers, HCW helpers, and CBO helpers from three CBOs in Duluth, Minnesota (Generations Health Care Initiatives, The Arc Northland, and Lincoln Park Children and Families Collaborative); and health researchers from the Essentia Institute of Rural Health. Twenty‐two individuals were recruited to the CAT by the aforementioned CBOs through a health equity key informant network, and 18 accepted.

CAT members were invited to participate in all six CBPE steps according to their interest and availability. All CAT members actively engaged in > 1 CBPE step. Meetings followed a structured format, and time was reserved at the end to encourage feedback, whether delivered in‐meeting or via an anonymous post‐meeting survey. Feedback was reviewed, acted on, and shared at the next meeting by the group's primary facilitators AWO, SN, MLH, and MR. Strategies to offset imbalances in structural power dynamics included sharing stories about why members were invested in the study's work; acknowledging group member differences in background, experiences, and social privilege; and deliberation over communication practices (e.g., promoting transparency) [30, 31]. Experts with lived experience who were members of the CAT were compensated > $44,000 for study work.

Resourceful and the CBPE study launched in July 2021. The study ended in January 2024 after 45 virtual and in‐person monthly sessions lasting 60–90 min each. If necessary, meetings with identical content or activities were offered at two different times each month to accommodate CAT members' schedules. Initial sessions were designed to build group trust, share how Resourceful worked, and discuss the structural, historical, and cultural factors related to the use and evaluation of Resourceful. The health researchers and experts with lived experience from Generations Health Care Initiatives met weekly to coordinate project steps and activities. Graphic summaries of the activities and outputs from each CBPE step are provided in the Supporting Information.

Step 1 of the CBPE process was completed by 16 CAT members between August 2021 and December 2021. The CAT identified areas most important to the seeker experience of finding and/or connecting to social care using Resourceful. Group consensus was built via a 3‐round modified Delphi Method (see Supporting Information for detailed description) to identify and describe intended experience “goals” for seekers when Resourceful was used [32, 33].

Step 2 was completed by 15 co‐authors between January 2022 and May 2022. Step 1 “goals” became the “intended results” of the logic model (i.e., a roadmap of the information, people, activities, measures, and other needs for evaluation) [34]. The CAT worked both individually and collectively to generate the logic model's “planned work” (i.e., resources and activities) needed to achieve the intended results (see Supporting Information for detailed description).

Step 3 was completed by 15 co‐authors between June 2022 and November 2022. The CAT used dot voting to identify the activities most relevant and important for achieving four identified goal areas [35], whereby each member placed up to four dot votes for each goal area (16 dots total) onto one or more of the activities identified in Step 2. The process yielded seven activities that were most important to explore.

Step 4 was completed by nine co‐authors between December 2022 and June 2023. Co‐authors A.W.O., M.L.H., M.R., and S.N. drafted initial interview guides based on Step 3, which were reviewed and modified by participating CAT members (interview guides available upon request) [36].

Quantitative data were obtained between March 2023 and April 2023 with a self‐administered REDCap survey following a modified Dillman recruitment method [37, 38, 39]. Survey invites were sent to 780 adult seekers and 38 HCW helpers at Essentia Health with valid email addresses who used Resourceful for social care activities between August 2022 and February 2023. Seekers who did not speak or read English, opted out of research at Essentia Health, or lacked legal decision‐making capacity to consent were excluded. After the initial invitation, up to three follow‐up reminder emails were sent once a week to non‐responders. Consented seeker participants were entered into a drawing for one of 10 $35 gift cards.

Qualitative data were collected by six co‐authors between May 2023 and June 2023 via semi‐structured phone interviews that were audio recorded and transcribed [40]. Interview invites were emailed to 19 seekers and 14 HCW helpers attached to > 1 closed‐loop Resourceful referrals who indicated a willingness to be interviewed on the study survey. Interview invites were also sent to individuals at the 12 CBOs attached to Resourceful referrals of seeker and HCW helper interview participants to comprehensively explore the perspectives of all involved in the closed‐loop referrals. Interviewers and study participants did not have an established relationship before the interview. Experts with lived experiences led all but one interview with participants after completing human subjects research training [41], and training from qualitative researchers AWO and MLH on semi‐structured interviewing and data trustworthiness techniques [42, 43]. A health researcher or expert with lived experience from Generations Health Care Initiatives was present at interviews to provide support (e.g., field notes or additional follow‐up questions). All interviewers introduced themselves when the interview began along with their study interest, role, and goal. Interviewers discussed observations, potential biases, and field notes immediately after the interview.

An interview guide collectively developed and pilot‐tested by CAT members participating in CBPE Step 4 organized interviews into three sections to understand: how participants used Resourceful; how Resourceful impacted the experience or success of finding or connecting seekers to help; and opportunities for improving Resourceful. Each section opened with a standard stem question, with all other follow‐up questions either spontaneously generated by interviewers or selected from a pre‐written list [36]. All seekers and CBO helpers completing an interview received a $35 gift card.

The study quantitatively analyzed 11 “CBPE Experience” variables representing the experience of seekers and HCW helpers using Resourceful to find or connect seekers to help (i.e., four goals identified in Step 2: Trust, Communication, Helpful Help, Seeker‐Centeredness; and seven activities identified in Step 3: Facilitate Rapport, Simple Plan, Engagement as Equals, Highlight Strengths, Privacy, Adequate Time, Discrimination). Variable measures used a single 5‐point Likert‐type scale to measure participant agreement with statements developed or adapted from the care experience literature by the CAT [44, 45, 46, 47, 48, 49, 50]. Additional data were collected on respondent demographics either known or thought likely by the CAT to be associated with social health inequities (age, gender, ethnicity/race, location). Seeker surveys had items for the number of dependents and areas of unmet HRSN in the past year (survey instruments available by request). The 11 CBPE Experience variables informed the collection and analysis of qualitative data. Additional qualitative variables were inductively generated using Braun and Clarke's thematic analysis methodology [51].

Step 5 was completed by five co‐authors between July 2023 and October 2023. Descriptive statistics were used to analyze survey data. Braun and Clarke's [51] six‐phase thematic analysis methodology was used to analyze qualitative data. AWO trained three co‐authors K.G., S.N., and S.W. in the thematic analysis process.

Step 6 formally ended in January 2024 after study funding expired, although dissemination activities continued. All CAT members were invited to propose and participate in dissemination opportunities as well as given opportunities to object or express concerns. This article represents one example of dissemination approved and co‐developed by the CAT [52].

### 2.1 IRB Review

This study was reviewed and approved by the Essentia Health Institutional Review Board.

## 3 Results

The CAT identified 11 areas most important to informing metrics for evaluating the seeker experience finding and/or connecting to social care using Resourceful. These 11 areas are identified and operationally defined in the first column of Table 2. Four of the 11 areas represented seeker experience outcome goals intended to be achieved through the use of resourceful: (1) strengthened TRUST among seekers and helpers; (2) improved COMMUNICATION among seekers and helpers; (3) seekers received more HELPFUL HELP from helpers; and (4) enhanced SEEKER‐CENTEREDNESS in helper care approaches. Seven of the 11 areas represented activities deemed integral to achieving these goals: (1) FACILITATING RAPPORT between seekers and helpers; (2) co‐developing SIMPLE PLANS for seekers; (3) seeker and helper ENGAGEMENT AS EQUALS; (4) helpers HIGHLIGHT STRENGTHS of seekers; (5) helpers protecting seeker PRIVACY; (6) ADEQUATE TIME for seekers and helper encounters; and (7) guarding against DISCRIMINATION.

**TABLE 2:** Comparison of Community‐based participatory Evaluation (CBPE) Experience Variables between Seekers and Healthcare Worker Helpers.

Experience variables	Somewhat or strongly agree		Δ
	Seekers (*n* = 62)	Helpers (*n* = 14)	Seeker% minus helper%
TRUST (Use of Resourceful demonstrated HCW helpers had the seeker's best interests at heart)	66%	86%	‐20%
COMMUNICATION (Use of Resourceful enabled HCW helpers to better understand the seeker and what they need to be healthy)	61%	57%	4%
HELPFUL HELP (Use of Resourceful assisted HCW helpers reduce the time and energy seekers would have otherwise spent finding or connecting to services)	56%	93%	−37%
SEEKER‐CENTEREDNESS (Use of Resourceful by HCW helpers make seekers feel like a person and not just a number)	60%	79%	−19%
FACILITATING RAPPORT (Use of Resourceful made it easy for seekers and helpers to talk about finding and/or getting connected to help)	68%	86%	−18%
SIMPLE PLAN (Use of Resourceful made it easy for seekers to understand how to find and/or connect to help)	59%	86%	−27%
ENGAGEMENT AS EQUALS (Use of Resourceful made seekers engage as equals with helpers when making decisions for finding and/or connecting to help)	54%	64%	−10%
HIGHTLIGHT STRENGTHS (Use of Resourceful enables helpers to highlight seeker strengths in the process of finding and/or connecting to help)	42%	57%	−15%
PRIVACY (Use of Resourceful maintained seeker confidence that their privacy was protected throughout the process of finding and/or connecting to help)	66%	64%	2%
ADEQUATE TIME (Use of Resourceful provided enough time for seekers to share information they felt was important to find and/or connect to help)	53%	50%	3%
DISCRIMINATION^a^ (Use of Resourceful led to seekers being treated worse than others because of what they shared for finding and/or connecting to help)	5%	21%	−16%

^a^ The DISCRIMINATION variable was reverse coded.

Abbreviation: HCW, healthcare worker.

Of the 818 people who received survey invitations, 62 seekers (8% response rate) and 14 HCW helpers (37% response rate) provided usable responses. Table 3 describes the demographic characteristics of participating seekers and HCW helpers, as well as the HRSNs that seekers sought social care for in the previous year. The average age of all respondents was 47.6 years, and respondents predominantly self‐identified as white persons and female. About half of seekers resided in rural/sparsely populated areas (53%) and reported > 1 dependent (47%). The most common areas of social care needs/services reported were financial assistance (31%), behavioral health (29%), food insecurity (23%), and disability services (23%). Of 33 seeker and HCW helper respondents invited to interview, 11 completed interviews.

**TABLE 3:** Demographics and social care services sought by survey respondents and interviewees.

Variable	Health system	Survey respondents		Interviewees	
	(*n* = 1.1 M)	Seekers (*n* = 62)	HCW helpers (*n* = 14)	Seekers (*n* = 6)	HCW helpers (*n* = 5)
Age (years)					
≤ 17	20%	—	—	—	—
18–24	9%	4	0	0	0
25–34	12%	9	2	1	1
35–44	12%	14	3	2	1
45–54	11%	6	3	0	1
55–64	12%	14	4	2	2
65–74	13%	9	0	1	0
75–84	8%	1	0	0	0
≥ 85	3%	0	0	0	0
Prefer not to say/no response	—	5	2	1	0
Race/ethnicity					
American Indian/Alaska Native persons	3%	5	1	1	1
Asian persons	1%	2	1	0	1
Black/African American persons	3%	2	0	0	0
Hispanic/Latinx persons	3%	2	0	0	0
Middle Eastern/North African persons	< 1%	0	0	0	0
Native Hawaiian/Pacific Islander persons	< 1%	0	0	0	0
White persons	87%	50	13	4	5
Other persons	< 1%	0	0	0	0
Prefer not to say/no response	—	1	0	2	0
Gender					
Female	49%	43	9	4	3
Male	51%	8	4	1	2
Non‐binary/prefer not to say	—	11	1	0	0
Residence (RUCA code)					
Urban (RUCA 1–3)	—	30	4	3	4
Nonurban (RUCA 4–10)	—	32	9	3	1
Prefer not to say/no response	—	2	1	0	0
Dependents (#)					
0	—	33	—	5	—
1	—	11	—	0	—
2	—	4	—	1	—
3	—	6	—	0	—
4+	—	4	—	0	—
Social care needs/services sought in the past year					
Behavioral health services/counseling	—	18	—	1	—
Emergency assistance	—	10	—	1	—
Dental care	—	13	—	3	—
Disability services	—	14	—	2	—
Food shelf/pantry/helpline	—	14	—	3	—
Financial assistance	—	19	—	4	—
Home health services	—	4	—	1	—
Homelessness services/housing assistance	—	8	—	0	—
Insurance/other coverage application assistance	—	9	—	1	—
Medical assistance/MN medicaid program	—	11	—	1	—
Transportation services	—	12	—	1	—
Utility assistance programs	—	11	—	3	—
Other	—	6	—	1	—

*Note*: Respondents were able to select more than one response option for the variables of “Race/ethnicity” and “Social Care Needs/Services Sought in the Past Year.” Internal health system did not collect or report on variables of “Gender: non‐binary,” “Residence,” “Dependents,” and “Social Care Needs/Services Sought in the Past Year.”

Abbreviations: HCW, healthcare worker; M, million; MN, Minnesota; RUCA, rural–urban commuting area codes.

Participant agreement with statements from the 11 CBPE Experience variables are described in Table 2. Most seeker and HCW helpers agreed on some level that using Resourceful positively impacted 10 of 11 CBPE Experience variables. Percentage differences in the level of agreement between seekers and HCW helpers for each item ranged from 2% to 37%, with “ensuring helpful help” representing the largest difference and “protection of seeker privacy” the smallest. Seeker and HCW helper levels of agreement differed by ≥ 10 percentage points for eight of the 11 CBPE Experience variables. HCW helpers reported higher levels of agreement than seekers for seven of these eight variables.

**FIGURE 1:**
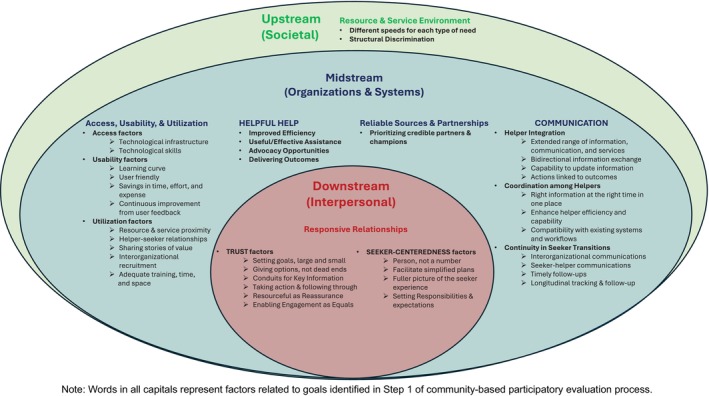
Thematic Map of Factors Driving the Seeker Experience of Social Care with Resourcefulness.

Of 33 survey respondents (seekers = 19, HCW helpers = 14) involved with > 1 closed‐loop Resourceful referrals who were willing to be interviewed, 11 completed interviews (seekers = 6, HCW helpers = 5). An additional 12 CBOs involved in a closed‐loop referral were also sent interview invites, of which representatives from two CBOs completed an interview. Six hours of interviews with seekers, HCW helpers, and CBO helpers (mean: 26 min, 14 s per interview) were transcribed, including data from two closed‐loop referral triads (seeker+HCW helper+CBO helper attached to same referral). Thematic analysis produced 89 codes applied to 282 excerpts and refined into 15 final codes organized under six themes: Resource/Service Environment; Platform Access/Usability/Utilization; Helper Integration/Coordination/Continuity; “Helpful Help”; Reliable Sources/Partnerships; Responsive Relationships (Figure 1). Themes and codes informed a 3‐level thematic map of factors affecting the experience of individuals using Resourceful for social care: downstream (interpersonal‐level factors within or adjacent to seeker‐helper encounters), midstream (organizational‐level factors within or adjacent to institutions/systems organizing services), and upstream (societal/structural‐level factors like legislation and workforce dynamics) [53, 54, 55, 56]. Table 4 presents quotes representative of each theme and its subcodes.

**TABLE 4:** Themes, codes, and representative quotes of factors driving the seeker experience of social care with resourceful.

Theme (level)	Code	Representative quotes
Responsive Relationships (Downstream/Interpersonal)	*Trust* Factors related to whether Resourceful facilitates strengthened trust between seekers and helpers. Examples include circumstances, factors, or influences that contribute to helpers using Resourceful: to set appropriate goals with seekers for addressing their unmet needs (i.e., setting goals, large and small); to provide seekers with options, not dead ends when confronting unmet needs; as a conduit for collecting key information about seeker strengths and assets; to build credibility with seekers by taking action and following through; to provide reassurance that Resourceful can be used if times get tough; and to enable engagement with seekers as an equal.	“I think it makes a big difference when somebody comes in and gives options. When I'm working with [a seeker], I'm not going in to tell them, ‘Okay, you screened positive for food. This is what you need to do.’ I go in there and I present what I have. I explain what Resourceful is and give them the option to search on their own or I can help them and we can work together to do it. Really, it puts the ball in their court, gives them the power, and makes them feel like they're part of the process, rather than—I know a lot of the time people state that they feel like they're being told what to do by people or, ‘Oh, if you have this, you have to call this number. They'll help you.’ And that's as far as it goes. Whereas, Resourceful, it's more of a collaboration between me and the patient and we get to work together. We get to kind of explore in a sense. We're going to refer to these programs. And we're going to see which ones work, which ones didn't. And it's a team effort…It's probably stating what I said, but just to make sure. I think it's just that letting people basically search and see these programs explaining what Resourceful is and just letting them navigate on their own and just really lets them just kind of, I don't know, feel like they're a huge part of the process.”—Healthcare Worker Helper “I was a little leery of like, how would they be able to help me with stuff like [unmet social needs] when they're my doctor's office? Would a doctor's office really know community resources? So for me, I was a little leery about that. The reason [I] wasn't so trusting that this person would be able to help me with stuff like transportation or food or resources. Or if I was, and now I'm not, if I was in a domestic violence relationship, help with that kind of stuff. But I see it as a medical model, kind of like your doctor's office. How would they know how to help me with that kind of stuff? I would think that's more like [county name], or the government, or something like that. So that's why there was a little bit of a trust level difference. Because at first I was kind of like, “I don't know if this is even—what is the point of answering these questions?” And then when it did help me, I was like, ‘Oh, wow. I'm so grateful’…So right now I don't have anything that I need help with. But it definitely did change when they did help me, and told me how to [use Resourceful], and sent me a letter in the mail, and gave me information. And it was a really helpful experience. So I definitely have a level of trust now.”—Seeker/Patient “Some people, I think, are embarrassed that they're having these troubles. And sometimes you ask all these questions right upon enrollment, and they really don't know you that well yet, so we review those questions again in 6 months, so oftentimes their answers will be different because we've built that relationship of trust. And sometimes the patients have actually called us and said, ‘You know,’ or, ‘I'm having trouble getting to my appointments, or things like that.’ We do a monthly touch base with them, so we also reach out once a month, and that's how we build our relationships.”—Healthcare Worker Helper “Now that I know how to [find and connect to help from a community‐based organization with Resourceful], I know what my options are, and I got the help from the Resourceful people … I understand how to call, who to call, and that that's even out there. Medical transport for somebody in a wheelchair is possible, and how to even do it. Because I had switched insurance companies and I didn't even know what do you do. I wasn't even sure, I'm in a wheelchair, how does this work? Yeah. It was a good experience. I'm glad I did it.”—Seeker/Patient
	*Seeker‐centeredness* Factors related to whether Resourceful helps seekers feel like a person and not just a number. Examples include circumstances, facts, or influences that contribute to the use of Resourceful to facilitate simplified plans that reflect the seeker's capability to understand and act on steps to effectively and efficiently address their unmet needs; helpers using the platform to improve their wholistic understanding of the seeker's preferences, values, and needs as they relate to finding and connecting to help (i.e., fuller picture of the seeker experience); and helpers formulating, sharing, and supporting seekers with what needs to done and what seekers can anticipate will happen when Resourceful is used (i.e., setting responsibilities and expectations).	“[The HCW helpers] they actually know me, and I'm not just a number. So again, that helps with the rapport, helps with me feeling comfortable when I discuss, especially really personal things, with my physicians. And with that—that ties into Resourceful—is having that rapport, having that connection and feeling like I am a person instead of just the stereotypical like, ‘Here's a brochure, figure it out.’ They actually take the time to walk you through what Resourceful was, how I could use it, and if I had any questions. So it wasn't just like, ‘Here's a brochure for more information.’ It was actually more of a, ‘Let's talk about this and get you the information you need.’”—Seeker/Patient “And in the room, we go over everything. And we talk about everything. So they have that time to really express what their needs are, what their concerns are. But then, while they're searching, and if they take this and search on their own, that gives them the freedom to do it whenever they have time and go home and search in the evenings or on a break from work if they are working. It gives them that time to be able to search, but spending that time with me as well, they have that extra, whether it's 15 min they want, a half hour. I've sat with people for 3 h. And they can really, really express their needs so we can go over it and we can narrow it down into, ‘These would be good areas to start searching here. Let's look into this. Do you want to search these or should I get the process going and start sending you information and you can pick and choose between what I send you?’”—Healthcare Worker Helper “…When we first are introduced. We spend about a half hour gathering information, what their needs are, what they've tried, what has worked, what hasn't…And see if they want to work together…A lot of the time when that confusion occurs, it's when [seekers] going on their own. I will sit in the room with them and kind of explain to them what Resourceful is. And that's part of the conversation we have as we're deciding what help, what they've tried. I'll ask them, ‘Is this something you want to work on together? Or do you want me to show you this program and explain what it is? And you can search and then reach out to me with any questions you have.’”—Healthcare Worker Helper “Yeah, I have a few patients that they just kind of want the convenience of, just give me the resources, you make the calls for me, you do this for me. You know what I mean? It's just kind of on me, which is okay. I just have some patients who just say they just kind of want things done for them…Like I said, life happens. They've got 87 other things going on and they don't have the time out of the day to make those phone calls. And that's perfectly fine.”—Healthcare Worker Helper
Access, Usability, and Utilization (Midstream/Organizations and Systems)	*Access* Circumstances, facts, or influences that contribute to a seeker's ability, or in some cases the ability of the helpers working on the seeker's behalf, to navigate to the Resourceful platform. Examples include the technological infrastructure and devices available to Resourceful users; and accommodating for Resourceful users' general knowledge, ability, and familiarity in using technology (i.e., technological skills).	“I think the accessibility of it is really helpful to them. The patients that I work with, often they consent to having those resources being sent to them via text message. And that's great because they just have it sent directly to their phone, and we all know in this day and age, everybody is on their phone. So just to have that right there, sent to them, and they can even go back to it if they need to, I think that's what makes it most useful.”—Healthcare Worker Helper “So some of the patients that I work with, they're older, they don't use text messaging or they don't have access to their email or they're not using the MyChart through Essentia Health. So typically what I do with those patients is I'll just go into Resourceful myself, kind of make a little list of resources I think that would help them, and then write down the contact information and then I give it to them over the phone or whatever.”—Healthcare Worker Helper

*Usability* Circumstances, facts, or influences that contribute to a seeker's ability, or in some cases the ability of helpers working on the seeker's behalf, to effectively and efficiently use Resourceful to find and connect to help once the platform has been accessed. Examples include assessing the rate of a seeker or helpers' progress in gaining experience or mastery in using Resourceful (i.e., learning curve); seeker and helper perceptions of how intuitive and easy to use the platform is; seeker and helper perceptions of how much time, effort, or expense is saved in using the platform; gathering and incorporating feedback from users to identify what's going well as continuously improve the tool's usability.	“It's during the interview process when we're talking about the needs and figuring out what they want. As we're searching programs, I can kind of look into programs that work for them and see what they've worked with, what they haven't. But then at that same time, I can kind of slide the keyboard and the mouse over their way and assess as they're searching. I'm like, ‘Why don't you look, and we'll kind of search together.’ I can see if they're able to navigate that system very well…at that point I can decide, maybe this is something I should continue to send referrals for or this person is 100% capable of doing this on their own. I'm just going to check in with them in a week.”—Healthcare Worker Helper “The resources were in a central location online. And then it seemed like it was pretty catered to my specific needs and eligibility. And again, just the wide variety of resources that were available. It was really convenient instead of having to go search for these things. And again, I feel like I have a good background of resources or knowing different resources. And some of those that were offered were interesting. I hadn't even thought about them or had any experience in knowing of those tools. So it was really interesting. Again, seeing how many resources are available and having it all in one location so I could easily connect with folks to dig into it a little bit more.”—Seeker/Patient “There have been patients who have reached back out to me saying, you know, thank you so much, this resource was so helpful. I was able to get assistance for A, B, and C. I've had a few patients reach out and say that they were able to get the certain foods that they needed for their special diet due to their heart condition. So I have gotten the feedback, it's just, I wish I could get it more, you know.”—Healthcare Worker Helper “I am actually really happy with the way things are going. It seems to be going very smooth. I like the meeting process. I like the working with the patient and the follow‐up. I would say about the only thing that I have concerns about from a patient searching on the Resourceful would be that Claimed Program. And I know it says on there, it has that little description, but I don't think many people really pay attention to that. So I think that—I'm not sure how to make that more apparent or more obvious when a plan is claimed and when it's not. I would say that's about the only complaint I have with Resourceful is that section there.”—Healthcare Worker Helper

*Utilization* Circumstances, facts, or influences that contribute to seekers' actual use of Resourceful, or in some cases the helpers working on the seeker's behalf. Examples include whether there are resources and services close enough to allow for practice and effective use by seekers (i.e., resource and service proximity or convenience); the strength and state of the connections between the seekers and helpers who would use Resourceful (i.e., seeker‐helper relationships, sharing stories of value, interorganizational recruitment); and Resourceful users' having an adequate amount of training, time, and places for helpers to work with seekers in addressing seeker unmet needs.	“I spoke with a family who was having some food insecurities in their home. And they said that they have been utilizing the food pantries that they felt like they needed a little bit more, just anything like that. [CBO name], they assist patients with the snap benefit program as well. So when I was able to talk with the patient about Essentia Health being partnered with this platform called Resourceful and being able to place referrals directly to these programs on the patient's behalf, they said that would be great because it would be more convenient if they were able to just get that phone call and say, this is what I'm looking for, this is the kind of assistance I need. And if they're able to provide assistance, that's great.”—Healthcare Worker Helper “I wasn't aware of [Resourceful] until I talked to my doctor. And I guess I'm not sure. I just feel like I didn't know that that stuff was available. And with that email I sent to her, she said it was a community outreach. I believe it was what it's called. She said, “Did you want me to get in touch with somebody?” And I said yes. And so like I said, I had no idea that that was available. That's why I just wanted her to understand that I'm struggling.”—Seeker/Patient “So we're using [Resourceful] to—I've even reached out and met with lots of community resources and educated them about this to send their patients to us because we're using Resourceful and we're connecting. And if they're not on Resourceful, ‘Get on Resourceful. Let me show it to you.’”—Healthcare Worker Helper “But the more effective tool it becomes for me, the more I have to learn more about it too. So I think a yearly training and yearly orientation is probably good in my situation, just to know kind of what capability has been added, or knowing more about it, so.”—Healthcare Worker Helper
Communication (Midstream/Organizations and Systems)	*Helper integration* Circumstances, facts, or influences that contribute to the fusion of data components from different organizations (e.g., health systems, community‐based organizations). Examples include an extended range of information, communication, and services on Resourceful than would otherwise be available to seekers and helpers; ability of seekers and helpers to frequently exchange and update information on the platform; and capacity to understand how actions on Resourceful affect outcomes desired by seekers and helpers (i.e., actions linked to outcomes).	“So let's say they tried to reach out to WIC, to get connected with WIC. And they missed their phone call. I can use Resourceful to go in and send out another referral and then add in the notes, ‘Patient missed the phone call. Patient is ready to connect,’ or call the office and let them know while the patient is there in real‐time. (Or I can) call them and say, ‘Hey, I have a patient here who has been trying to connect with you. She's ready to be connected again,’ or, ‘They are ready to be connected again.’ And then if the patient's with me, they can consent to leaving a number’…let's say they come back for another visit or I send myself a reminder to call the patient the following week to say, ‘Did they get connected with a public health service? Did they get signed up for WIC? Were they having any issues or struggles? Did they have any concerns about a breast pump or a car seat, or?’ And then in Resourceful, you can go in and add notes and change it from—the status, you can update it to pending or patient got help or patient declined or whatever the option might be.”—Healthcare Worker Helper “I find a program that is new every single day that I haven't heard of, that I didn't even know existed. And, yeah, so it's a daily occurrence that I'm finding something new and learning…I think just finding programs that are unexpected that I had no idea even existed.”—Healthcare Worker Helper “I think the tool has a login thing. So I could go in and kind of keep track of resources that I actually had connected with. There's ways where you can kind of show like, ‘Yeah, I was eligible for this,’ or, ‘No, I was not,’ or had all the contact information.”—Seeker/Patient “If [Essentia Health] didn't have Resourceful, I think [the information about my social needs] would've been just filed away.”—Seeker/Patient
	*Coordination among helpers* Circumstances, facts, or influences that contribute to the ability of these service organizations to effectively find, arrange, and use this data in service provision. Examples include how well Resourceful can provide helpers with the right type and amount of information in one place when it is most relevant for their intended use; enhances the efficiency and capability of helpers in the process of finding and connecting seekers to help; and how well the use of Resourceful is compatible with existing systems and workflows used by helpers.	“…[Resourceful] makes it easier for providers and nurses to—rather than put an order in, print it, fax it, it's streamlined. You can just do it through Resourceful. And then you can see it in the patient's chart. You can see that, yes, this referral is new. And then we started adding to it. So if, let's say, we're talking to—to give an example, the St. Louis County Minor Parent Program, they added a spot to refer. So we can now go through Resourceful to send that referral for teens, pregnant teens. And then we just kept adding. We would say to someone, ‘Have you seen Resourceful?’ It can be used by anybody, but it's an organization that we would utilize in Essentia Health. We ask them to look into Resourceful. It is streamlining our ability to improve the prenatal care altogether. It's really improved it…”—Healthcare Worker Helper “Well, [the helper who used Resourceful] told me somebody would contact me, but in my experience, I never have somebody—I never had anybody tell me, ‘Somebody will contact you,’ and that afternoon, somebody had already contacted me. I almost fell over. I mean, this was taken care of right away. It was not put in my file never to be looked at again.”—Seeker/Patient “…And so it's good to have those updates of what has taken place a lot of times. And so seeing that and reading that information is good because, number one, it alleviates some paperwork. It alleviates kind of some steps as far as phone calls and paperwork and fax machines, and things like that. And then seeing the outcome of how it's been handled and what's taken place so far. So yeah, those are all things that are efficient and can add to a person's proficiency in what they do, so.”—Healthcare Worker Helper “When I first started [at Essentia Health], we were only working with three community partners. And I had a really big patient list I was working with and only having three community partners was really tough because they didn't always help with the financial needs or the transportation needs. So having Resourceful and this whole listing of everything, it's so great. So I appreciate it.”—Healthcare Worker Helper
	*Continuity in seeker transitions* Circumstances, facts, or influences that contribute to the seeker experiencing an easy and seamless transition in services and communication from one organization to another. Examples include the degree and capability of helpers from different organizations to effectively exchange the necessary, relevant, and useful information for helping seekers (i.e., interorganizational communication); the degree and capability of helpers to effectively exchange necessary, relevant, and useful information with the seekers when they are transitioning between helper organizations (i.e., seeker‐helper communication); the seeker's receipt of necessary, relevant, and useful information, actions, or reminders after an encounter with a helper at or before the time they need it (i.e., timely follow‐ups); and the ongoing collection of necessary, relevant, and useful information by seekers and related to finding or connecting seekers to help that enables status and progress to be tracked over time (i.e., longitudinal tracking and follow‐up).	“I'm in a wheelchair. And I didn't know how to get transportation to medical appointments. And your person [used Resourceful to help me] find medical transportation and get coordinated with [health plan organization name] to get my doctor to sign off on a waiver thing that I need a lift, to get special transportation for myself because I'm in a wheelchair. To help me get to and from medical appointments. It was very, actually, very helpful.”—Seeker/Patient “So if I contact the patient, and say I sent them resources for food assistance, I give them a 7 day window. Just to give them time because life happens, you know. Just to give them time to update themselves and everything like that. So after that, the week after, I put or send the resources to the patient, I gave the patient a call or another MyChart message just to say, hey, we spoke last week, I sent you resources for food assistance. Were you able to get in contact with anybody or receive assistance? Do you need any further help?”—Healthcare Worker Helper “So then I show them, “Well, let's look and see what resources are out there for disabled veterans. So let's see what kind of help we can get you guys to support you.” So we went through and looked up a bunch of DA things, and it was there versus just googling it. And then letting them know, ‘When you come back in, I'll make a note in your appointment desk to meet with me. And let's just check in and see have you connected with them.’ And then I can update it in Resourceful so that their providers also know, ‘Oh, wow. They're getting this help. That's fantastic,’ right?”—Healthcare Worker Helper “For my own practical purposes, I think the way it could benefit me more is to pay more attention kind of to the follow‐up piece of it and how once a referral has been made to a resource following that pattern or following how it gets handled. In other words, right now, I know that there are some organizations where the referral is made online, and they will give you updates along the process. And then there's some resources on Resourceful that will just say, ‘Here's what the patient or your client needs to do to follow up on this referral.’ So it kind of puts it in their hands a little bit. I think it's always good to just kind of get a refresher of what new has been added. What things it can do. What all the capability that it has. And so it's not always easy to find the time to do that.”—Healthcare Worker Helper “Well, I think that's probably more where Resourceful comes in more proficiently, I guess. Because as you work with a person over time, over a period of time, there are more things that come out, more and more things that they may need resources for. So it's a continuous option to be able to access as you work with a person or a patient longer over a longer period of time. So there's just more scenarios that those opportunities to use Resourceful are available at that point.”—Healthcare Worker Helper
Helpful Help (Midstream/Organizations and Systems)	*Improved efficiency* Circumstances, facts, or influences that contribute to Resourceful being used to maximize support for seekers getting help to address their unmet needs with minimum wasted effort or expense.	“Resourceful helps simplify some things [for seekers and helpers]. And I think that the fewer hoops that you have to jump through, or the fewer barriers or call this number and they tell you to call another number, that's better. And if Resourceful can identify the needs when a client contacts them, and then direct that referral out to the appropriate agency, I think it's just helpful for the client that needs care.”—Helper at a Community‐Based Organization “I think [seekers/patients] are very happy that there's a tool that can help network so that it also helps them to—it also helps them to—it also helps them to realize how many things are out there and what things are redundant, what things might not be helpful, but it's a condensed search. You're not searching the whole web. You're searching specific to this.”—Healthcare Work Helper “…It makes us more efficient, plus we're able to track and follow up with patients, which gives them a much more improved level. At least these are the responses that I'm hearing from the patients. They feel much more cared for, and they feel supported, and they're getting connected to things that make their stress levels go down.”—Healthcare Worker Helper

*Useful/effective assistance* Circumstances, facts, or influences that contribute to helper activities perceived by the seeker to provide meaningful assistance in finding or connecting to help that addresses their unmet needs.	“…I have been to three ER visits in the injection in my spine last year at the hospital, they told me they have a community cares program. And the community care, I applied for that, and I got it, and I would've never done that if Resourceful wouldn't have helped me. And then when I got the bill for 950, they told me I could apply for it again and I said, ‘I'm getting an income tax return, I'll pay it.’ Because I don't want to use that service if I don't absolutely need it…Oh, no. She had to have seen from Resourceful because there's no way she would've known that. I'm sure that when Essentia Health called and said, ‘These are the things this person needs,’ and then she called me—because she gave me things I didn't even know about, and I would've never ever known about if I wouldn't have filled out that Resourceful.”—Seeker/Patient “Because we have done follow‐up with organizations where it could be we've connected the patient to housing, to getting into a minor or young parent housing and out of a negative situation. We've talked with them through the whole follow‐up. We've connected the parent program up in northern [county name]. And the responses, it's fantastic. It feels like it's closing—I have seen Resourceful used as a tool for where I can help close the gap between.”—Healthcare Worker Helper

*Advocacy opportunities* Circumstances, facts, or influences that contribute to helpers directly reaching out to other organizations on behalf of the seekers to resolve any confusion or discrepancies experienced by the seeker when their referrals are rejected.	“[The helpers] were fantastic. Matter of fact, they went over with me, as far as my SNAP benefits, and they told me—which I did contact social services. They told me that the limitations for the SNAP rules had changed. Back in September, they had raised. So I should have been getting more. And I did contact social services with that. But they say they have a formula…they're definitely an advocate for me. Not where like social services here in town, they're not advocating for me. They don't care. That's just how I feel. They just don't care.”—Seeker/Patient “…If people need help, and they need the help that I'm able to offer, then having somebody who's already sitting right in front of them saying, ‘Yeah, let me send your information to [CBO name] and they'll get in touch with you.’ First, it takes some confusion out of it because people don't always know what resources are available to them. So having something like that is really helpful. And then, also, taking the burden off of them to reach out to all these different places to try to get the help that they need. When it gets sent to us, then we get in touch with them. And that certainly makes it easier for them. And that is something that I've experienced, even in different places when you put it on them to make that contact, many times they don't. Whereas if they can say like, ‘Hey, let me get your contact information, and I'll get in touch with you,’ then it's more successful because it just makes their life simpler. And I mean, if they are hungry, then they probably already have a lot on their—a lot going on in their life to try to add another thing to do.”—Helper at a Community‐Based Organization

*Delivering outcomes* Circumstances, facts, or influences that contribute to Resourceful being used to effectively address the unmet needs of seekers and results in desirable health and well‐being outcomes as defined by seekers and helpers.	“I would have to try to rely on [CBO name] for transportation, and sometimes it was—at one point, when they were short on drivers, it was super hard to get reliable transportation to appointments. And if they were late, then I was late to my appointment, which would then make me miss my appointments. And I have a lot of complex medical problems that I need help with. And to have access to transportation through my insurance [which was discovered using Resourceful], and not even know at the time if that was something that was possible, really changed my life. Because it's a lot more reliable.”—Seeker/Patient “Working with a patient, they brought up they need help with their well. I had no idea that there was even wells in [city name] still. I thought this was a thing out in the country outside of town, but [the seeker's] well had collapsed. I thought, well, this will be a challenge. Let's start looking into some stuff [on Resourceful] and, within a minute, we found somebody that specifically dealt with wells…And, after all was said and done, it took them about 4 days. And they set up a plan where they completely re‐dug and set her up an entire new water well for $5 a month.”—Healthcare Worker Helper “…We can also check things like—connect them with, say, Milk Moms so they can get a blood pressure cuff and check in to see what their insurance covers, but then using Resourceful as tracking it. And then that data will also help us figure out, ‘Is that helping outcomes? Are people's blood sugars, blood pressures lower?’ We know that stress can be difficult on our health, right? So if we can lower those stressors that we know for a fact, evidence‐based practice that we know affect our health, those social determinants, if we can decrease that with support, like a wraparound support and networking through all of these different communities.”—Healthcare Worker Helper
Reliable Sources and Partnerships (Midstream/Organizations and Systems)	*Prioritizing credible partners and champions* Organizations and individuals listed on or who use Resourceful in high quality and consistent way that benefits seekers.	“I have seen some, where Resourceful has caused a little bit of frustration with people. Just simply with the Claimed Program portion. I think a lot of people get confused about, if they refer themselves, they assume that every one of those programs is going to reach out to them, which isn't the case. I don't think there's a real clear description of what that little check mark up in the corner means for a patient. So I think that kind of causes a barrier because they select seven or eight programs. Well, only one or two may reach out. But they don't know that four or five of those programs that they selected aren't Claimed Programs so they wouldn't reach out. They're informational pages only. So I think that can create a little bit of a barrier because people get discouraged…You can see when people get discouraged when they think that organization is just choosing not to reach out to them or that maybe they're at full capacity so they just don't follow through anymore.”—Healthcare Worker Helper “Usually when I'm working with the patient and I'm having them kind of search Resourceful as well, I like to first find out, ‘What was the experience like? Were they contacted? How did they reach out? Did they choose to call or did they use the—did they refer themselves?’ So, really, when the patient's using Resourceful, I try to gather information about how it works just so I know. So when I'm referring people to programs, I know how that program is going to respond to the patient.”—Healthcare Worker Helper
Resource and Service Environment Factors (Upstream/Societal)	*Different speeds for each type of need* Societal circumstances, facts, or influences that make it so different types of unmet needs (e.g., food insecurity, housing insecurity, financial assistance) require different amounts of time, effort, and expense to obtain.	“…they're getting what they need. Especially with food resources, housing is tough, they get the information they need, but it's a wait. It's just—a long wait. And I think everybody understands that so the expectations, typically thinking that (they're not) going to get an apartment in the next week or so. But when it comes to food and emergency financial assistance, things like that, getting connected to the resources and getting the help they need within, sometimes up to a month, But typically within a week to 2 weeks.”—Healthcare Worker Helper “…but from what I've been hearing from my counselors and doctors and such, is that if you come up onto one of these housing lists, like an apartment becomes available, one of the first questions they're going to ask you is, “Have you done your SPDAT?” Because that opens up a lot more doors in terms of fundage and whatnot. But it's also hard to get one done…”—Seeker/Patient “…usually, people, they understand that a lot of these programs are—they have limited resources. It's not instantaneous. If you reach out to a program, they're not going to call you the next day and say, ‘Hey, here's $200.’ So if I set those expectations and let them know it is a process. It's going to be—you're going to fill out applications, but this is where we're going to start. I think that they are extremely pleased with the outcome as long as they understand that it won't be an instantaneous process.”—Healthcare Worker Helper
	*Structural discrimination* The restriction of opportunity for individuals with a certain characteristic due to outlooks, rules, or practices that take place at the societal level.	“Well, that was not so much transportation as a cost to—none of these food shelves are located near me, so they're all an hour, an hour‐and‐a‐half hour away. And with the cost of gas, I'd be driving an hour‐and‐a‐half, and then sitting there for a couple hours waiting for free food. And the cost of—and then a lot of the free food was intended for huge families, like a lot of people. I can't eat all that food. And then I've spent all this money on gas to get there; it just did not make sense. I just decided I'd rather not eat at all. And, plus, for me, with my pain, the driving is what exacerbates the pain. So I'm in a car, and then I'm sitting someplace for 2 h. I just said—I tried several different food shelves in different locations, and I just said [inaudible]. And then I have to carry these big boxes into my house that are heavy stuff that—and then I had to throw a lot of it away because I can't consume that much. So this isn't working for me. I'd rather just not eat and just buy groceries when I can.”—Seeker/Patient “I think it would be useful to have more resources for seniors. Our program is Medicare patients, and we typically focus on 70 and older.”—Healthcare Worker Helper

Abbreviation: HCW, healthcare worker.

## 4 Discussion

This study used a CBPE approach to explore the experiences of patients using a CRRS platform with closed‐loop referral functionality to find and/or connect to social care. Quantitative survey findings revealed differences between seekers and HCW helpers in 11 quantitative experience variables potentially important to individuals and organizations for improving social care activities. Qualitative analysis of semi‐structured interviews identified factors underlying quantitative findings. Further evaluation produced 23 actionable takeaways informed by the literature for improving the experience of individuals using CRRS in social care.

### 4.1 Seeker‐Helper Comparisons

Survey results from seekers and HCW helpers suggested that using Resourceful positively impacted seeker experiences finding and/or connecting to help. However, the magnitude and type of impact on the experience noticeably differed between seekers and HCW helpers. Seeker and HCW helper responses were separated by ≥ 10 percentage points for eight of the 11 variables. HCW helpers overestimated Resourceful's positive impact on the seeker experience on all eight variables (Trust, Helpful Help, Seeker‐Centeredness, Facilitating Rapport, Simple Plan, Engagement as Equals, Highlighting Strengths, Discrimination), signifying potential perception differences in seeker needs and expectations in social care informed by CRRS. The discrepancy may indicate suboptimal experience outcomes from breakdowns in seeker‐helper relationships (i.e., downstream/interpersonal level) [57, 58, 59], missed opportunities for care quality improvements (i.e., midstream/system‐level) [60, 61], and exacerbation and reinforcement of structural inequities (i.e., upstream/societal‐level) [62].

### 4.2 Seeker Experience Factors: Themes and Takeaways by Level

Qualitative analysis identified factors of potential importance for understanding and improving the experience using CRRS in social care from the perspective of seekers and helpers. The following sections discuss the implications of these factors at the level where they predominantly occur (i.e., downstream, midstream, upstream) and 23 corresponding actionable takeaways potentially important for improving the seeker experience informed by the literature.

### 4.3 Downstream

The responsive relationships theme represented the downstream level, drawing together two factors tied to interpersonal needs and expectations of individuals using resourceful in social care: strengthening trust and enhancing seeker‐centeredness. Both themes are consistent with evidence about the importance of helpers, who serve as human navigators when CRSS are utilized [63]. First, trust was a pre‐requisite for a relationship being responsive to a seeker's preferences, values, and needs in social care. Trusting relationships were built over time and appeared to facilitate feelings of security (i.e., mitigated fear of judgment, information privacy concerns), seeker empowerment (i.e., seeker self‐efficacy and autonomy), collaboration (i.e., open dialog, joint decision‐making), and credibility (i.e., believable, competent, reliable). This finding aligns with emerging evidence about the importance of trust in health system‐mediated social care. The absence of trust is one explanation for why seekers screening positive for unmet HRSNs may decline assistance [64]. A seeker's level of trust in a clinician or health system may facilitate or impede them from sharing sensitive HRSN information [64]. Thus, a positive seeker experience with a helper using CRRS may strengthen relationships and credibility or weaken them if seeker needs, expectations, and concerns are not addressed. Actionable takeaways helpers can use to potentially strengthen trust with seekers include utilizing active or deep listening, using common language, recognizing seekers' lived experience expertise, acknowledging seeker strengths, and providing emotional support [65, 66, 67, 68, 69, 70]. Helpers can also view closed‐loop referral information from CRRS as indicators of a breakdown in need of intervention and not solely an endpoint for an analysis or dashboard summary [71].

Second, seeker‐centeredness was essential to using Resourceful to build a responsive relationship characterized by helpers pursuing a full and up‐to‐date picture of the seeker's needs to ensure clarity in expectations, plans, and roles. For example, one seeker with food insecurity may prefer a referral to a food shelf along their bus route, while another may want an out‐of‐town resource to avoid seeing people they know. Needs and help preferences of seekers may also change over time. For example, a seeker feeling overwhelmed or in crisis may prefer to delegate all activities for getting assistance to helpers (i.e., a referral is sent at the seeker's instruction from one helper to another, with the helper receiving the referral reaching out to the seeker rather than the other way around). However, after the crisis is successfully managed, the seeker may feel empowered to independently find and/or connect to help. From the seeker perspective, both instances may be the most appropriate use of CRRS, even if neither fully utilized all closed‐loop functionality. Capturing these nuances with measures in CRRS reports may be important given emerging evidence that care personalization and quality are key mechanisms for improving health and well‐being [64, 70, 72, 73]. Actionable takeaways for potentially enhancing seeker‐centeredness include the use of shared decision‐making to tailor goals and plans (e.g., access to healthier food, evaluating options) [74] and incorporating individual‐level social care experience measures into CRRS reports.

### 4.4 Midstream

Midstream‐level factors were represented by four themes: access, usability, and utilization of resourceful; communication; helpful help factors; and reliable sources and partnerships. Each theme captured important elements threaded throughout the qualitative data generated in the interviews.

The access, usability, and utilization of resourceful theme constituted the three terms in its name. Access refers to factors affecting the abilities of seekers and/or helpers to navigate to resourceful. According to helpers interviewed, access was challenging for seekers in rural/sparsely populated areas with limited Internet or cell phone coverage, as well as for seekers with trouble operating computers and smartphones. However, this did not prevent helpers from checking in with seekers periodically or sharing new relevant resources by text or other means that might be useful to seekers in the future. Usability refers to factors affecting the ability to use Resourceful effectively and efficiently, which differed for seekers and helpers. Helpers cited needs for sufficient time, training, and workplace space to use Resourceful. Most seeker comments indicated a dependence on a helper's ability to assess (e.g., evaluate technological literacy, incorporate feedback for process/system enhancements), support (e.g., tutorial and practice sessions), and highlight (e.g., share stories of learning and/or success) a seeker's use of Resourceful to find and/or connect to help. The findings align with other studies demonstrating the importance of human navigators with adequate training, resources, and reasons to adopt CRRS in social care activities [63, 75, 76]. Seekers and helpers liked that resourceful streamlined information, actions, and status updates in one location better than internet search sites and created potential for a more tailored experience. Access and usability factors were necessary for resourceful adoption and utilization, but insufficient without seekers and helpers also holding two additional views. First, resourceful needed to be perceived as more useful than their default ways of finding and/or connecting to help (e.g., faster, less cumbersome, filled a HRSN program niche or gap in knowledge). Second, seekers and helpers had to see resourceful as having staying power. Seekers and helpers also suggested that positive word‐of‐mouth reviews and success stories promoted utilization, which was consistent with the literature [16, 71, 76]. Actionable organizational‐level takeaways were the development of a baseline standard workflow for helpers, offering recurring training and best practice newsletters, accommodating time and space needs for CRRS use, acting on seeker and helper usability feedback, and amplifying success stories to grow intra‐ and inter‐organizational tool awareness and utilization [16, 77, 78].

The Communication theme represented factors tied to integration and coordination among helpers, and continuity in seeker transitions. Helper integration was the capability to extend the range and utility of information between and within organizations as well as with seekers (e.g., programs awareness, eligibility, referral‐related actions, outcomes). Some helpers noted Resourceful enabled prevention and/or recovery from referral process breakdowns (e.g., follow‐up after a missed phone call) and identification of opportunities for system improvement. Coordination among helpers represented factors enabling seekers and helpers to find, organize, and use information on resourceful. Several helpers mentioned how Resourceful helped make it easier to find, share, and act on information quickly, resulting in more time and knowledge to improve meaningful use of the tool and better continuity in seeker transitions (i.e., seeker experiencing interactions across different helper organizations as coherent and connected) [44]. Examples included perceptions by seekers that helpers were sending, receiving, and using information that minimized question repetition, fast response times, and evidence of longitudinal tracking. Actionable organizational‐level takeaways are exploring collaborative performance approaches for aligning intra‐ and inter‐organizational workflows involving CRRS‐mediated social care (e.g., eliminate avoidable time/effort burdens for order entry/printing/faxing) [79] and deliberately reallocating new capacities to activities optimizing the seeker experience (e.g., progress notifications for seekers, documenting seeker concerns after closing the referral) [80].

The Helpful Help theme represented system‐level factors for resourceful's capability to reduce the time and energy that seekers and helpers otherwise spent finding and/or successfully connecting to help. Interviewee comments pertained to the tool's efficiency and effectiveness in getting help, increased helper capability to advocate for or remove seeker burdens, and ultimately deliver a positive outcome. For study participants, “helpful help” contrasted with “help” in that the latter can be any level of assistance (e.g., list of programs) while the former focuses on whether and how much activities remove barriers, redundancies, and stress while augmenting the seeker‐recognized benefits of (e.g., completing program applications, acquiring required documentation, scheduling an appointment and corresponding transportation). “Helpful help” via Resourceful allowed helpers to advocate for seekers intra‐ and inter‐organizationally (e.g., highlight the connection between social and medical needs) rather than putting all the burden and responsibility on the seeker. “Helpful help” bridges potential discrepancies between seeker‐recognized health and well‐being improvements and successful CRRS closed‐loop referrals. Actionable organizational‐level takeaways for “helpful help” when using resourceful begin with more detailed documentation and tracking of the seeker's self‐perceived needs as well as specific helper action to make seekers feel cared for, less stressed, and more hopeful [64]. Emerging evidence suggests that acknowledging and addressing these areas are important to seeker health and well‐being independent of fully addressing unmet social needs [70, 81, 82].

The reliable sources and partnership theme represented system‐level factors seekers and helpers identified as important to ensuring the credibility and quality of using Resourceful in social care. Some helpers indicated that unanswered referrals exacerbated anxiety, stress, and frustration in seekers, especially those seekers newly using Resourceful without a helper. In these instances, seekers may blame both the organizations sending and receiving the referral. Some helpers were concerned about referring to new or unfamiliar organizations without a sense of their service quality. Actionable organizational‐level takeaways include adopting policies and procedures favoring referrals to CBOs with quality service records along with cautioning seekers of potential non‐responses and establishing corresponding plans for follow‐up and evaluation of their experiences. Helper organizations can also focus on maximizing the quality over the quantity of key partnerships with CBOs (e.g., explore incentive alignments or cost‐sharing offered by states) [16, 71, 76, 80, 83, 84, 85].

### 4.5 Upstream

The resource and service environment theme represented upstream‐level factors. Interviewees highlighted the importance of setting seeker and helper expectations for finding and/or connecting to help across different need areas accompanied by a discussion of the structural forces behind them. Societal factors are often complex and difficult for individuals to notice in everyday life, let alone understand or explain (e.g., housing displacement from gentrification [86], technological inequities from digital redlining [87], health risks from climate change [88]). Actionable takeaways include recognition and acknowledgment by seekers and helpers that unmet HRSNs often extend to forces beyond their individual control (e.g., fewer resources and infrastructure in rural/sparsely populated areas, bias toward marginalized groups) [89], CRRS may not be the best tool for every context or situation, and that CRRS can reflect and be prone to perpetuating structural discrimination without larger scale advancements in health equity. Helper organizations can ensure there are other resources beyond CRRS available to support seekers with unmet HRSNs (e.g., 211) [90, 91] and deploy tools that best fit seeker needs and preferences.

### 4.6 Limitations

The findings and corresponding interpretations should be moderated according to the study's limitations. First, non‐response and volunteer bias may have skewed results from surveys and interviews, especially for seekers. Data to compare demographic and other factors of survey respondents and non‐respondents were not available at the time of writing. However, the percentage differences between survey respondent samples and the health system's service area were < 6% for race/ethnicity and age variables, although females were overrepresented in the survey sample by > 20%. Furthermore, individuals in crisis actively trying to meet their basic needs would be less likely to participate, while those seekers who benefited from using Resourceful to address a past crisis might be more inclined to participate. Consequently, the results may overrepresent responses from individuals greatly helped via Resourceful. Similarly, the seekers and helpers who rated Resourceful most negatively in the survey did not wish to be interviewed about their experiences, which limits the understanding of the challenges experienced by this population when Resourceful was used. Second, unintentional cues for responses from study participants desired by those conducting the semi‐structured interviews are a risk for this type of data collection. Thus, the CAT worked together to create prompts and questions that were neutrally phrased as well as having the two investigators in each interview monitor each other for instances of this bias when the other was speaking. Bias from non‐verbal messages was also mitigated through the use of phone‐only interviews, although this strategy also prevented investigators from capturing non‐verbal cues from interviewees. Third, the transferability of the findings may be limited to integrated health systems in rural‐facing communities with similar demographic profiles. In particular, individuals using a CRRS outside of an integrated health system in areas serving populations that greatly differ in proportion for racial/ethnic, age, gender, urban density, and other demographic characteristics (see Table 3 and Supporting Information) may report other distinct experiences.

Fourth, interview responses from participants were assumed to be accurate and truthful of their experience without transcripts being returned for corrections or comments. Finally, several biases were introduced from the 11 CBPE Experience variables. For example, each variable was tied to only a single item with high face validity judged by the CAT. Furthermore, the a priori identification by the CAT of these 11 variables that were carried over to thematic analysis may have overshadowed codes or themes that otherwise would have been identified by study participants.

### 4.7 Future Research

Future research should focus on the transferability of the findings about seeker views, preferences, and expectations for CRRS tools like Resourceful to communities demographically and geographically distinct from this study to expand our understanding of the influence of these factors on the seeker experience. Additional operational field testing and participatory implementation science activities are needed to further explore potential discrepancies in seeker and helper perceptions of the former's experience with CRRS and develop corresponding best practices to address those discrepancies that may lead to suboptimal outcomes [92, 93].

## Supporting information


**Data S1.** Supporting Information.
