# Supplementary material for: Getting Inside Closed‐Loop Referrals: Exploring the Patient Experience of Finding and Connecting to Social Care With a Community Resource Referral System Using a Community‐Based Participatory Approach
Source: Health Serv Res. 2025 Feb 16;60(Suppl 3):e14451. doi: 10.1111/1475-6773.14451 (PMC12052525; doi:10.1111/1475-6773.14451)

**SUPPLEMENTARY MATERIAL**

**Additional Details of Survey Respondent Demographics**

The mean age of seekers was 47.7 years with a standard deviation of 15.9. The mean age of healthcare worker (HCW) helpers was 47.4 years with a standard deviation of 9.5. Eighty-one percent of seeker respondents self-identified as white. Ninety-three percent of HCW helper respondents also identified as white. Among seeker survey respondents, 70% self-identified as female, 13% self-identified as male, 7% self-identified as non-binary/third-gender, and 10% preferred not to say or didn’t respond to the item. Among helpers, 64% self-identified as female, 29% self-identified as male, and 7% did not respond. Persons self-identifying as black/African American, Hispanic/Latinx, Middle Eastern/North African, Native Hawaiian/Pacific Islander, or non-binary gender were absent from qualitative data. Persons outside of a of 25-74 year age range, or who sought out homelessness or housing assistance services in the past year were absent from qualitative data)

**Additional Details of each Community-based Participatory Evaluation (CBPE) Step**

*CBPE Step 1: Use of adapted Delphi Method to Name and Frame Goals*


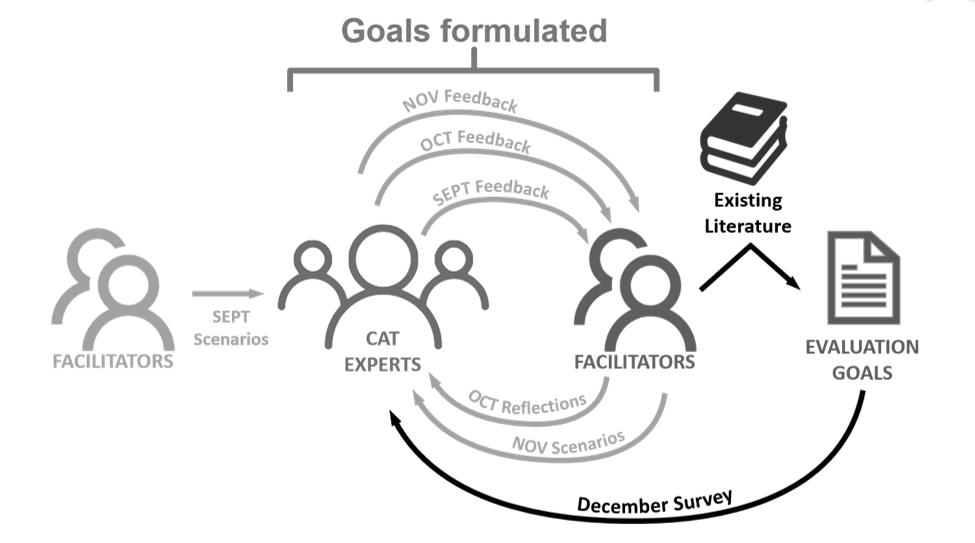


The modified Delphi Method began with three facilitators (co-authors AWO/MR/SN) developing and presenting to the CAT experts (co-authors AC;EK;HM;JK;KG;MG;NA;RSJ;SB;SW and acknowledged contributors SD;TG; SG;YA) with case scenarios of common situations where Resourceful might be used. Case scenarios were developed by facilitators as a figurative blank canvas framed with objective details (who, what, when, where, why) to maximize the opportunity for community advisory team (CAT) experts to apply rich descriptions based on their expertise, experiences, and stories. CAT experts would independently record their thoughts, then break into pairs to discuss, and finally convene as a large group to share their perspectives and reactions that were recorded by the health researchers. The notes were summarized and compared with existing literature by the facilitators, re-presented to CAT experts for validation, which then informed another round of the exercise. The process was repeated three times.

*CBPE Step 2: Develop a Logic Model for Success*


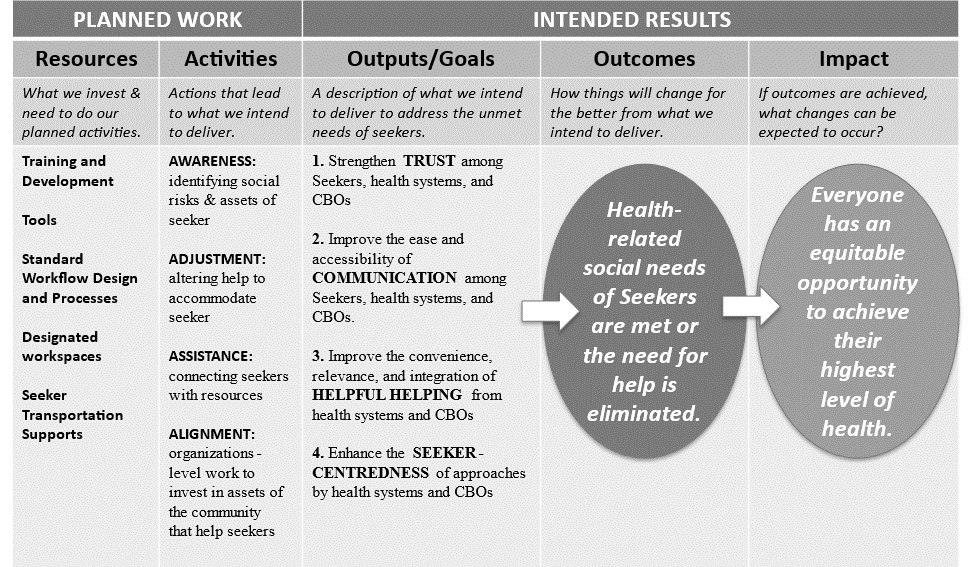


Step 2 was completed by coauthors AWO;EK;HM;JK;KG;KM;MG;MLH;MR;NA;RSJ;SB;SN;SW;TJ.

*CBPE Step 3: Focus the Evaluation*


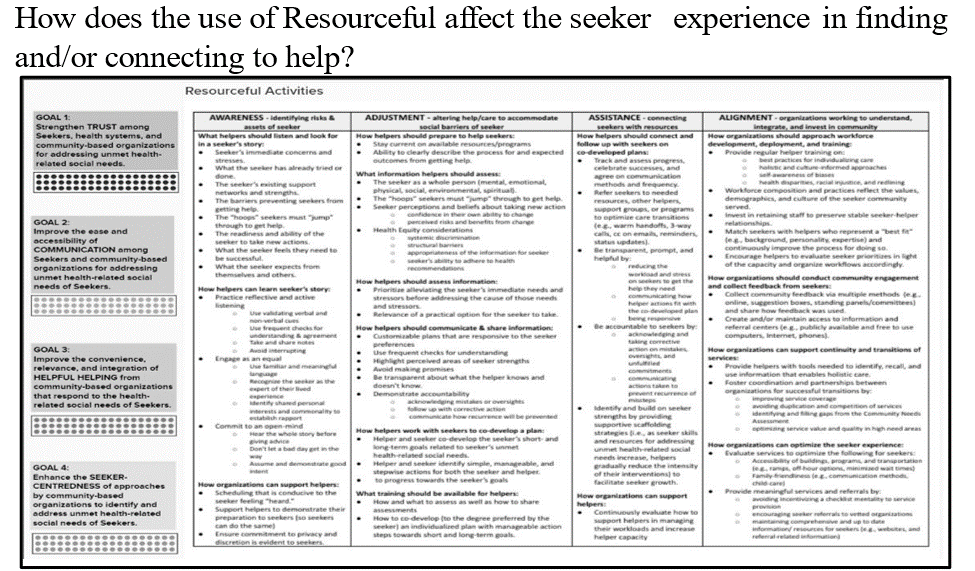


Step 3 was completed by coauthors AWO;EK;HM;JK;KG;KM;MG;MLH;MR;NA;RSJ; SB;SN;SW;TJ. Forty nine activity statements with corresponding resources were carried over from Step 2 and were organized into four groupings from the National Academies of Science, Engineering, and Medicine’s Social Care Framework: Awareness (identifying social risks/assets), Adjustment (altering clinical care for social barriers), Assistance (connecting/referring social care resources), and Alignment (understand, organize, and invest in social care).^11,33^

*CBPE Step 4: Gather Evidence*


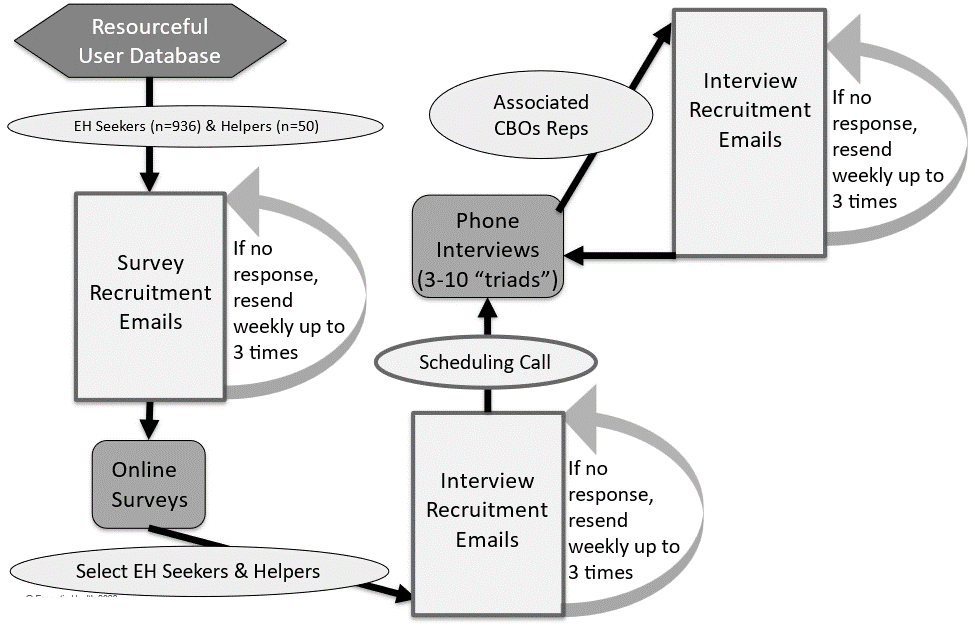


Step 4 was completed co-authors AE;AWO;KG;MLH;MR;NA;RSJ;SN;SW.

*CBPE Step 5: Analyze Information*


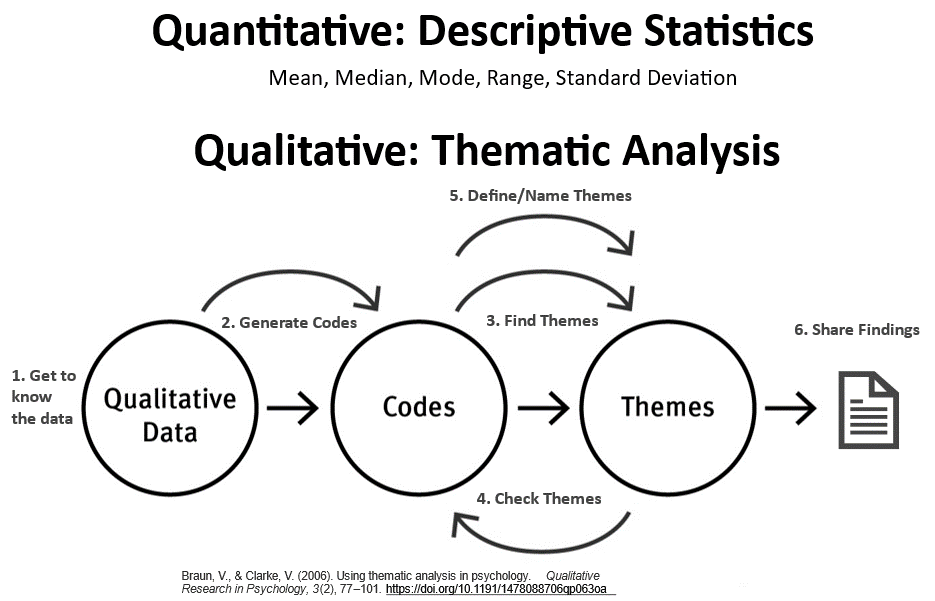


Step 5 was completed by six co-authors AWO/KG/MLH/SN/MR/SW.

1. *Get to know the data*: All coders independently read interview transcripts without taking notes to immerse themselves in the data, and then re-read each transcript a second time while writing down initial ideas.
2. *Generate codes*: Interview transcripts were converted by AWO into Microsoft Excel following a process created at Vanderbilt University to enable systematic classification and collation of interesting features in the data using a short name/code.^91^ Three transcripts were randomly selected for each coder to independently code for the 11 CBPE Experience variables while creating and defining inductive codes for features they found interesting in the data that did not fit within the CBPE Experience codes. The unit of analysis for coding was each continuous statement by a speaker and each unit could be assigned multiple codes. Coders then met to compare what they found and reconcile discrepancies. Once consensus was reached by coders the previous step was repeated for all remaining transcripts, with three transcripts coded per round.
3. *Find themes*: All coders independently grouped codes from the previous step into potential themes using poster boards. The themes were meant to capture what coders felt connected data to reveal important insights and meaningful patterns that were not apparent at surface level. The coders then met to identify and resolve inter-coder differences as well as generate an organizing structure that told “a multi-faceted story of the data.”^50^
4. *Check themes*: The coding group worked together to ensure themes were internally coherent, consistent, and distinctive in relation to each other and the original text that was coded. The result of this process was an initial draft of a thematic map.
5. *Define/Name Themes*: The coding group repeatedly met to iteratively refine the themes and their organizational positioning in the thematic map, including the names and operational definitions of themes. The process produced a thematic map representing data-supported factors driving the seeker experience in encounters where Resourceful was used.
6. *Share Findings*: The coding group worked together to select quotes, narratives, and illustrations that best exemplified themes in connection to the research question and intended audience.

*CBPE Step 6: Share and Celebrate Success*


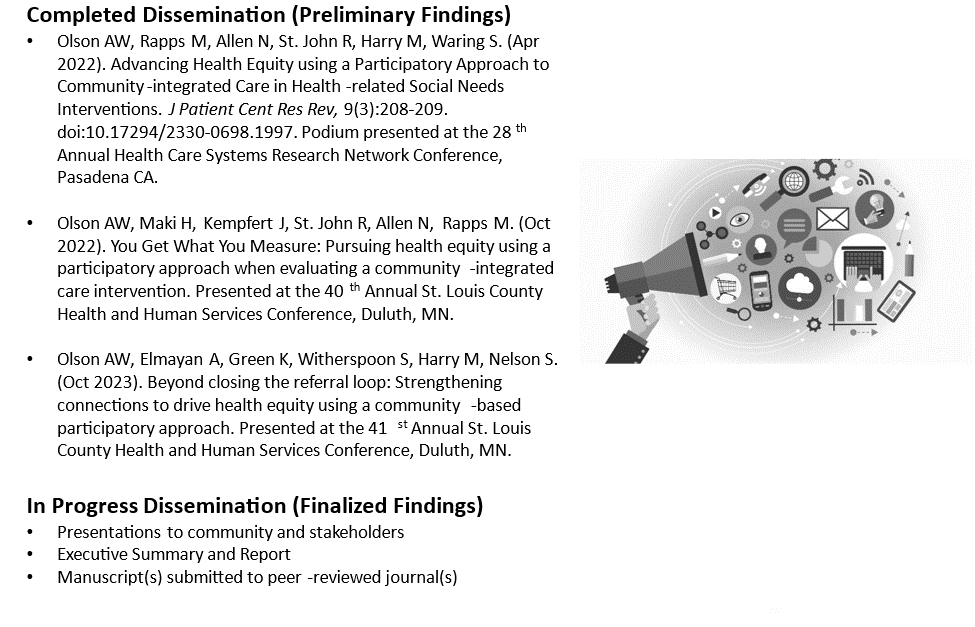

Supplement: Supplementary file 1 — Data S1. Supporting Information. [file HESR-60-0-s001.doc]
